# Nomogram predicting cesarean delivery undergoing induction of labor among high-risk nulliparous women at term: a retrospective study

**DOI:** 10.1186/s12884-022-04386-8

**Published:** 2022-01-21

**Authors:** Hang Zhou, Ning Gu, Yan Yang, Zhiqun Wang, Yali Hu, Yimin Dai

**Affiliations:** Department of Obstetrics and Gynecology, Nanjing Drum Tower Hospital, Nanjing University Medical School, 321 Zhongshan Road, Nanjing, 210008 Jiangsu China

**Keywords:** Induction of labor, Cesarean delivery, Nomogram, Internal validation, External validation

## Abstract

**Background:**

Our aim was to create and validate a nomogram predicting cesarean delivery after induction of labor among nulliparous women at term.

**Methods:**

Data were obtained from medical records from Nanjing Drum Tower Hospital. Nulliparous women with singleton pregnancies undergoing induction of labor at term were involved. A total of 2950 patients from Jan. 2014 to Dec. 2015 were served as derivation cohort. A nomogram was constructed by multivariate logistic regression using maternal, fetal and pregnancy characteristics. The predictive accuracy and discriminative ability of the nomogram were internal validated by 1000-bootstrap resampling, followed by external validation of a new dataset from Jan. 2016 to Dec. 2016.

**Results:**

Logistic regression revealed nine predictors of cesarean delivery, including maternal height, age, uterine height, abdominal circumference, estimated fetal weight, indications for induction of labor, initial cervical consistency, cervical effacement and station. Nomogram was well calibrated and had an AUC of 0.73 (95% confidence interval [CI], 0.70-0.75) after bootstrap resampling for internal validation. The AUC in external validation reached 0.67, which was significantly higher than that of three models published previously (*P*<0.05).

**Conclusions:**

This validated nomogram, constructed by variables that were obtained form medical records, can help estimate risk of cesarean delivery before induction of labor.

**Supplementary Information:**

The online version contains supplementary material available at 10.1186/s12884-022-04386-8.

## Background

Induction of labor is one of the most frequently used methods to initiate labor. In the United States, more than 22% pregnancies undergo induction of labor and almost one third of inductions will end in cesarean delivery [1].

The status of cervix has been recognized as one of the most important factors affecting the mode of delivery, and an unfavorable Bishop score (≤ 5) is the predominant risk factor of failed induction [2, 3]. Other studies have proposed that, several maternal and fetal characteristics, such as maternal age, parity, maternal height, body mass index, gestational age and fetal position, also affect the success rate of labor induction [4–8].

It has been widely assumed that induction of labor increases the risk of cesarean delivery. Previous studies have reported that cesarean delivery rates varied from 22.6 to 50% among nulliparous after induction of labor in different institutes, most of which included patients for both medical and non-medical indications [9–13]. However, one randomized trial has demonstrated that elective induction can decrease risk of cesarean delivery among low-risk nulliparous [14]. For high-risk nulliparous, indications for labor induction, such as diabetes mellitus and hypertension, have been reported to associate with cesarean delivery [10].

However, there is little knowledge about how to evaluate the risk so far, since these factors are seldom used in a comprehensive fashion. Therefore, it is reasonable to develop and validate a nomogram of cesarean delivery for nulliparous undergoing induction of labor, by combination of maternal, fetal and pregnancy characteristics.

## Materials and methods

The protocol of the retrospective study was approved by the Ethic Review Committee of Nanjing Drum Tower Hospital (reference number 2020-027-01, date of approval 2020-02-25). Written informed consent was obtained from all women enrolled in the program. All nulliparous with singleton, term, cephalic pregnancies who underwent induction of labor from Jan. 2014 to Dec. 2016 at Nanjing Drum Tower Hospital were enrolled.

Data on maternal and neonatal characteristic were abstracted from medical record. These data were double-checked by two obstetricians. Women with cervical dilation ≥3 cm were excluded since they might have been in spontaneous labor and were misclassified as induction of labor. Besides, women with missing data were also excluded for further data analysis. Factors enrolled for analysis were 1) maternal demographic characteristics, including maternal age, maternal height, maternal weight at delivery, uterine height and abdominal circumference; 2) medical indications for induction of labor, including premature rupture of membrane, late term, diabetes mellitus (gestational diabetes and pregestational diabetes), hypertensive disorder of pregnancy, liver dysfunction, fetal growth restriction and oligohydramnios; 3) obstetric conditions, including gestational age, fetal position, cervical examination at admission (cervical dilation, effacement, position, consistency and station), Bishop score; and 4) neonatal characteristics, including estimated fetal weigh, neonatal weight, neonatal sex. Results of PROBAAT trial and further meta-analysis has demonstrated that cesarean delivery rate was similar between Foley catheter group and prostaglandin group [15]. Besides, the original purpose of the retrospective study was to provide a user-friendly tool for both doctors and patients. The procedures of induction might be too professional and make patients confused. Therefore, methods of induction were not included in the study.

Gestational age was determined by the last menstrual period and confirmed by ultrasound examination. Timing to start induction was mainly based American College of Obstetricians and Gynecologists (ACOG) committee opinion “Medically indicated late-preterm and early-term deliveries” published in April 2013 [16].

The admission exam was the full 5 component Bishop score (cervical dilation, effacement, position, consistency and station). A Bishop score of <6 was considered unfavorable. All women with intact membrane whose Bishop score <6 received at least 1 method for cervical ripening: Foley catheter, vaginal misoprostol 25 μg every 4 h or vaginal dinoprostone (Propess). The standard application of Foley catheter has been established by our group previously and chosen as the first choice for cervical ripening for women without vaginal infection [16]. The choice of vaginal misoprostol or dinoprostone (Propess) was mainly based on provider preference. Artificial rupture of membrane was considered once the Bishop score ≥ 6.

Women who had successful vaginal delivery after induction of labor were classified as successful induction of labor; those who ended with cesarean delivery for any reason after induction of labor were classified as failed induction of labor. The model was constructed using derivation cohort from Jan. 2014 to Dec. 2015, which has been used to analyze cesarean delivery rate by 10-Group Classification System [17]. The dataset of Group 2a (nulliparous, single cephalic, ≥ 37 week, induced labor) had detailed variables for evaluation and perfectly fitted the target population in our research. Women with same inclusion criteria form Jan. 2016 to Dec. 2016 were enrolled as validation cohort. Continuous variables were presented as mean and standard deviation or median and interquartile range, where appropriate. Categorical variables were presented as frequencies and percentages. Student t test and Chi square test were used for continuous and categorical date in univariate analysis, respectively. All variables with a *P*<0.05 in univariate analysis were then included in a logistic regression model. Covariates were removed in a stepwise fashion until all covariates in the final value had a *P*<0.05. A nomogram was created based on coefficients weighted by the logistic regression model in R. The nomogram was internal and external validated by discrimination and calibration. Discrimination was assessed by receiver-operative characteristics (ROC) analysis using 1000 bootstrap resampling, and Calibration curve was graphically assessed by plotting the observed rates against the nomogram-predicted probabilities.

To compare the nomogram with other existing prediction model, we searched Medline between 1987 and 2020 on prediction model for induction of labor. The search strategy consisted of keywords of “induction of labor” and “prediction model”. Studies were selected in a two-stage process. Firstly, we went through title and abstract of all citations. Secondly, we obtained full reports published in English which established mathematical models to predict cesarean delivery after induction of labor, with sufficient detail to calculate the probability of cesarean delivery using our own data. Studies on both nulliparous and multiparous were also included. Area under the curve (AUC) for ROC analysis between the nomogram and models finally included were compared.

## Results

During 2014 to 2015, a total of 11,006 deliveries occurred at our hospital, of which 2961 met inclusion criteria. Due to incomplete data, 11 pregnancies were excluded. Therefore, 2950 pregnancies were finally enrolled as derivation cohort. A total of 1935 pregnancies admitted in 2016 were enrolled as validation cohort.

Table 1 showed the antepartum characteristics of pregnancies in derivation and validation cohort. The cesarean delivery rate slightly in increased from 13.2% in derivation cohort to 16.4% in validation cohort. PROM, consisted of over 40% of study population, remained the first indication for induction of labor, followed by late term, which consisted of 25% of the population. The derivation cohort and validation cohort shared similar characteristics in both maternal and fetal features.

**Table 1:** Antepartum characteristics of nulliparous undergoing induction of labor at term

Characteristic	Training Cohort *N* = 2950	Validation Cohort *N* = 1935	*P*
Maternal age (y)	28.4 ± 3.1	28.6 ± 3.0	0.016
Weight (kg)	71.0 ± 9.3	71.2 ± 9.1	0.431
Height (cm)	162.0 ± 4.5	161.9 ± 4.6	0.742
Uterine height (cm)	35.5 ± 2.2	35.4 ± 2.2	0.032
Abdominal circumference (cm)	101.3 ± 6.3	101.2 ± 6.1	0.780
Gestational age (wk)	39 (39-41)	40 (39-41)	0.007
Fetal sex			0.585
Male	1542 (52.3)	996 (51.5)	
Female	1408 (47.7)	939 (48.5)	
Estimated fetal weight (g)	3500 (3200-3600)	3500 (3200-3600)	0.270
Initial cervical dilation (cm)			0.002
0 (0 points)	2880 (97.6)	1913 (98.9)	
1-2 (1 points)	70 (2.4)	22 (1.1)	
Initial station			0.106
-3 (0 points)	962 (65.5)	583 (30.1)	
-2 (1 points)	1932 (32.6)	1322 (68.3)	
-1 or 0 (2 points)	56(1.9)	30 (1.6)	
+ 1 (3 points)	–	–	
Initial cervical effacement (%)			<0.001
0-30 (0 points)	5 (0.2)	2 (0.1)	
40-50 (1 points)	520 (17.6)	256 (13.2)	
60-70 (2 points)	2205 (74.7)	1492 (77.1)	
≥ 80 (3 points)	220 (7.5)	185 (9.6)	
Initial cervical position			<0.001
Posterior (0 points)	1015 (34.4)	570 (29.5)	
Mid position (1 points)	1875 (63.6)	1352 (69.9)	
Anterior (2 points)	60 (2.0)	13 (0.7)	
Initial cervical consistency			<0.001
Firm (0 points)	44 (1.5)	17 (0.9)	
Medium (1 points)	1495 (50.7)	1102 (57.0)	
Soft (2 points)	1411 (47.8)	816 (42.2)	
Bishop Score	5 (4-5)	5 (4-5)	0.621
<6	2185 (74.1)	1421 (73.4)	
≥ 6	765(25.9)	514 (26.6)	
Indications for induction of labor			<0.001
PROM	1319 (44.8)	941 (48.6)	
Late term (41 weeks or greater)	779 (26.4)	522 (27.0)	
Diabetes mellitus	472(16.0)	167 (8.6)	
Hypertensive disorder of pregnancy	81 (2.7)	108 (5.6)	
Others	299 (10.1)	197(10.2)	
Cesarean delivery	392 (13.3)	317 (16.4)	

Data are presented as mean ± standard derivation, number (%) or range

*PROM* Premature rupture of membrane

Maternal, neonatal and obstetric characteristics were compared between women who delivered vaginally and women who delivered by cesarean delivery by univariate analysis (Table 2). Detailed information about indications for induction of labor among women in derivation cohort was presented in Table 3.

**Table 2:** Univariate analysis of antepartum characteristics of nulliparous in train cohort

Characteristics	Vaginal Delivery *N* = 2558	Cesarean Delivery *N* = 392	*P*
Maternal age (y)	28.2 ± 3.1	29.4 ± 3.4	<0.001
Weight (kg)	70.7 ± 9.1	73.1 ± 10.4	<0.001
Height (cm)	162.1 ± 4.5	160.5 ± 4.4	<0.001
Uterine height (cm)	35.4 ± 2.2	36.2 ± 2.4	<0.001
Abdominal circumference (cm)	100.9 ± 6.1	103.6 ± 6.8	<0.001
Gestational age (wk)	39 (39-40)	40 (39-41)	0.003
Estimated fetal weight (g)	3500 (3200-3600)	3500 (3300-3700)	<0.001
Initial cervical dilation (cm)			0.025
0 (0 points)	2491 (97.4)	389 (99.2)	
1-2 (1 points)	67 (2.6)	3 (0.8)	
Initial station			<0.001
-3 (0 points)	783 (30.6)	179 (45.7)	
-2 (1 points)	1723 (67.4)	209 (53.3)	
-1 or 0 (2 points)	52 (2.0)	4 (1.0)	
+ 1 (3 points)			
Initial cervical effacement (%)			<0.001
0-30 (0 points)	2 (0.1)	3 (0.8)	
40-50 (1 points)	430 (16.8)	90 (23.0)	
60-70 (2 points)	1918 (75.0)	287 (73.2)	
≥ 80 (3 points)	208 (8.1)	12 (3.1)	
Initial cervical position			0.336
Posterior (0 points)	893 (34.9)	122 (31.1)	
Mid position (1 points)	1613 (63.1)	262 (66.8)	
Anterior (2 points)	52 (2.0)	8 (2.0)	
Initial cervical consistency			<0.001
Firm (0 points)	35 (1.4)	9 (2.3)	
Medium (1 points)	1256 (49.1)	239 (61.0)	
Soft (2 points)	1266 (49.5)	144 (36.7)	
Bishop Score			<0.001
<6	1853 (72.4)	332 (84.7)	
≥ 6	705 (27.6)	60 (15.3)	

Data are presented as mean ± standard derivation, number (%) or range

*P* values were calculated by Student t test or Chi square test, as appropriate

**Table 3:** Indications for induction of labor among nulliparous women

Indications	Vaginal Delivery *N* = 2558	Cesarean Delivery *N* = 392
PROM	1182 (46.2)	137 (34.9)
Late term (41 weeks or greater)	647 (25.3)	132 (33.7)
Diabetes mellitus	408 (15.9)	64 (16.3)
Hypertensive disorder of pregnancy	58 (2.3)	23 (5.9)
Others	263 (10.3)	36 (9.2)
Liver dysfunction	61 (23.2)	9 (25.0)
Fetal growth restriction	30 (11.4)	3 (8.3)
Oligohydramnios	172 (65.4)	24 (66.7)

Data are presented as number (%)

*PROM* Premature rupture of membrane

In univariate analysis, the following variables had a *P*<0.05 and were considered in logistic regression modeling: maternal age, weight, height, uterine height, abdominal circumference, gestational age, estimated fetal weight, initial cervical dilation, initial station, initial cervical effacement, initial cervical consistency and indications for induction of labor. After stepwise logistic regression analysis, independent risk factors associated with cesarean delivery after induction of labor were shown in Table 4. The following 9 variables remained significantly associated with cesarean delivery: maternal age, height, uterine height, abdominal circumference, estimated fetal weight, initial station, initial cervical effacement, initial cervical consistency and indications for induction of labor. For every 1-year increase in maternal age, there was a 9% increase in the odds of cesarean delivery (odds ratio [OR] 1.09, 95% confidence interval [CI] 1.05-1.13). Decreased maternal height was associated with increased probability of cesarean delivery (OR 0.91, 95%CI 0.88-0.93). A 1 cm increase in uterine height, 1 cm increase in abdominal circumference and 100 g increase in estimated fetal weight were associated with 6.0% (OR 1.07, 95%CI 1.00-1.13), 5.0% (OR 1.05, 95%CI 1.03-1.08) and 6.0% (OR 1.06, 95%CI 1.01-1.12) increase in the odds of cesarean delivery, respectively. Three components of Bishop score, including initial station, initial cervical effacement and initial cervical consistency were associated with risk of cesarean delivery. Initial cervical effacement levels 60-70%, 40-50% and 0-30% were associated with increased risk of cesarean delivery (OR 1.79, 95%CI 0.97-3.31; OR 2.19 95%CI 1.14-4.21; OR 9.23, 95%CI 1.26-67.56). Hypertensive disorder of pregnancy was one of the most important risk factors, since it had over a 2-fold risk of cesarean delivery (OR 2.38, 95%CI 1.38-4.13), followed by late term, which associated with 58.0% increase in the odds of cesarean delivery (OR 1.58, 95%CI 1.20-2.08).

**Table 4:** Independent risk factors for cesarean delivery among nulliparous women undergoing induction of labor at term

Characteristics	Unadjusted OR (95%CI)	*P*	Adjusted OR (95%CI)	*P*
Maternal age (y)	1.11 (1.07-1.14)	<0.001	1.09(1.05-1.13)	<0.001
Height (cm)	0.92(0.90-0.95)	<0.001	0.91 (0.88-0.93)	<0.001
Uterine height (cm)	1.17 (1.11-1.22)	<0.001	1.07 (1.00-1.13)	0.038
Abdominal circumference (cm)	1.07 (1.05-1.08)	<0.001	1.05 (1.03-1.08)	<0.001
Estimated fetal weight (100 g)	1.14 (1.09-1.19)	<0.001	1.06 (1.01-1.12)	0.013
Initial station		<0.001		<0.001
-1 or 0 (2 points)	Ref		Ref	
-2 (1 points)	1.58 (0.57-4.40)		1.26(0.44-3.62)	
-3 (0 points)	2.97 (1.06-8.32)		1.96(0.68-5.68)	
Initial cervical effacement (%)		<0.001		0.046
≥ 80 (3 points)	Ref		Ref	
60-70 (2 points)	2.59 (1.43-4.70)		1.79 (0.97-3.31)	
40-50 (1 points)	3.63 (1.94-6.78)		2.19 (1.14-4.21)	
0-30 (0 points)	26.00 (3.96-170.64)		9.23 (1.26-67.56)	
Initial cervical consistency		<0.001		0.006
Soft (2 points)	Ref		Ref	
Firm (0 points)	2.26 (1.07-4.80)		2.33 (1.06-5.11)	
Medium (1 points)	1.67 (1.34-2.09)		1.45 (1.14-1.83)	
Indications for induction of labor		<0.001		<0.001
PROM	Ref		Ref	
Late term (41 weeks or greater)	1.76 (1.36-2.28)		1.58 (1.20-2.08)	
Diabetes mellitus	1.35 (0.99-1.86)		1.03 (0.73-1.44)	
Hypertensive disorder of pregnancy	3.42 (2.05-5.72)		2.38 (1.38-4.13)	
Others	1.18 (0.80-1.75)		1.22 (0.81-1.85)	

*PROM* Premature rupture of membrane, *OR* Odds ratio

**Fig. 1:**
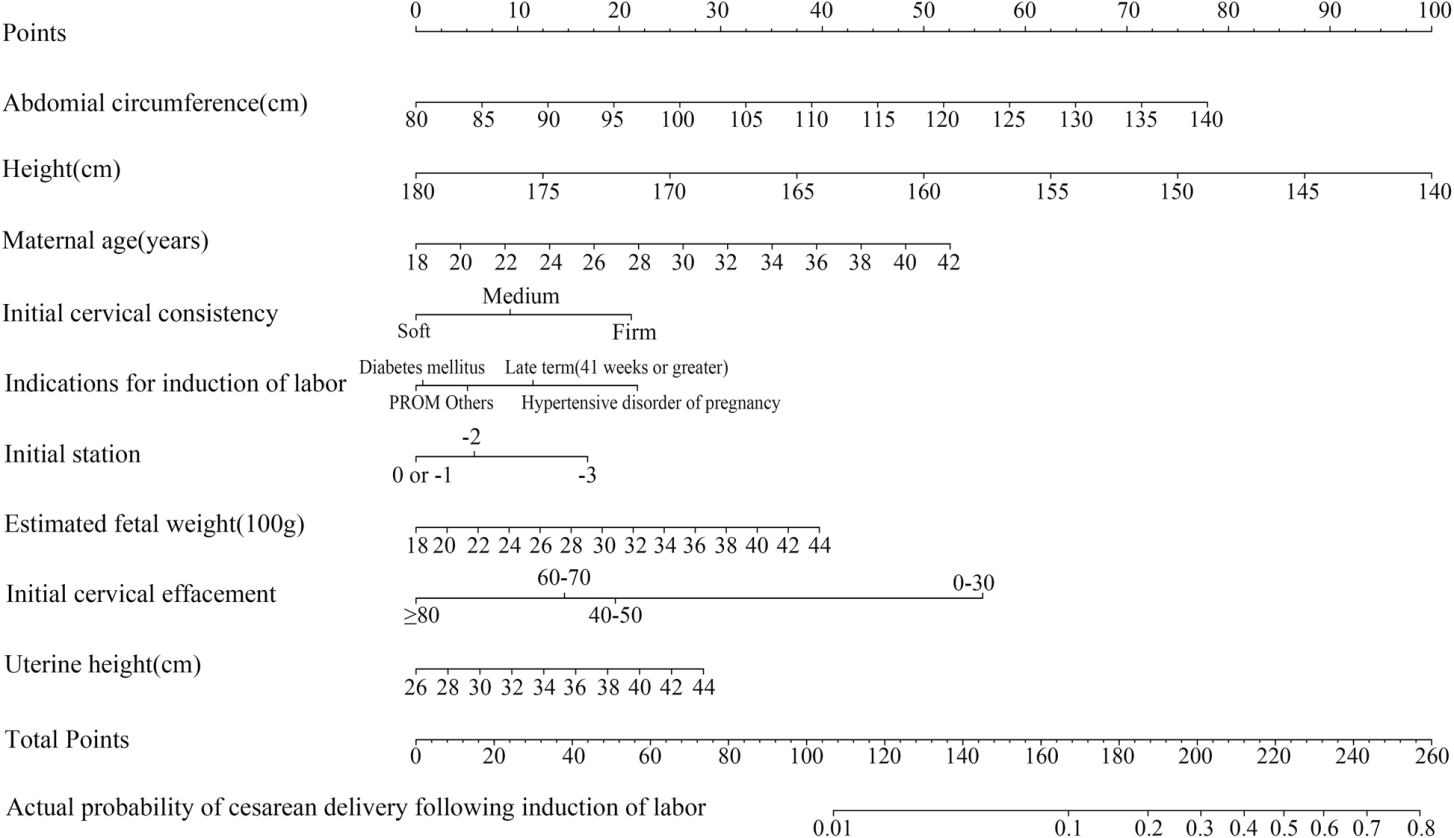
Nomogram predicting risk of cesarean delivery undergoing induction of labor among nulliparous women at term. For a given patient, points are assigned to each of the variables using the point axis at the top of the figure and a total score is derived. The total points correspond to a predicted probability of cesarean delivery after induction of labor at term. For example, a nulliparous woman of 30 years (26 points) with hypertensive disorder (22 points) is in her 37th week of pregnancy. Height of the patient is 155 cm (63 points), with 30 cm (6 points) of uterine height and 100 cm (26 points) of abdominal circumference, and the estimated fetal weight is 3000 g (18 points). The cervical condition is unfavorable [consistency, firm (21 points); station, − 3 (17 points); effacement (%), 0-30 (56)]. The total point reaches 255, with an estimated probability of cesarean delivery of 78% after induction of labor

**Fig. 2:**
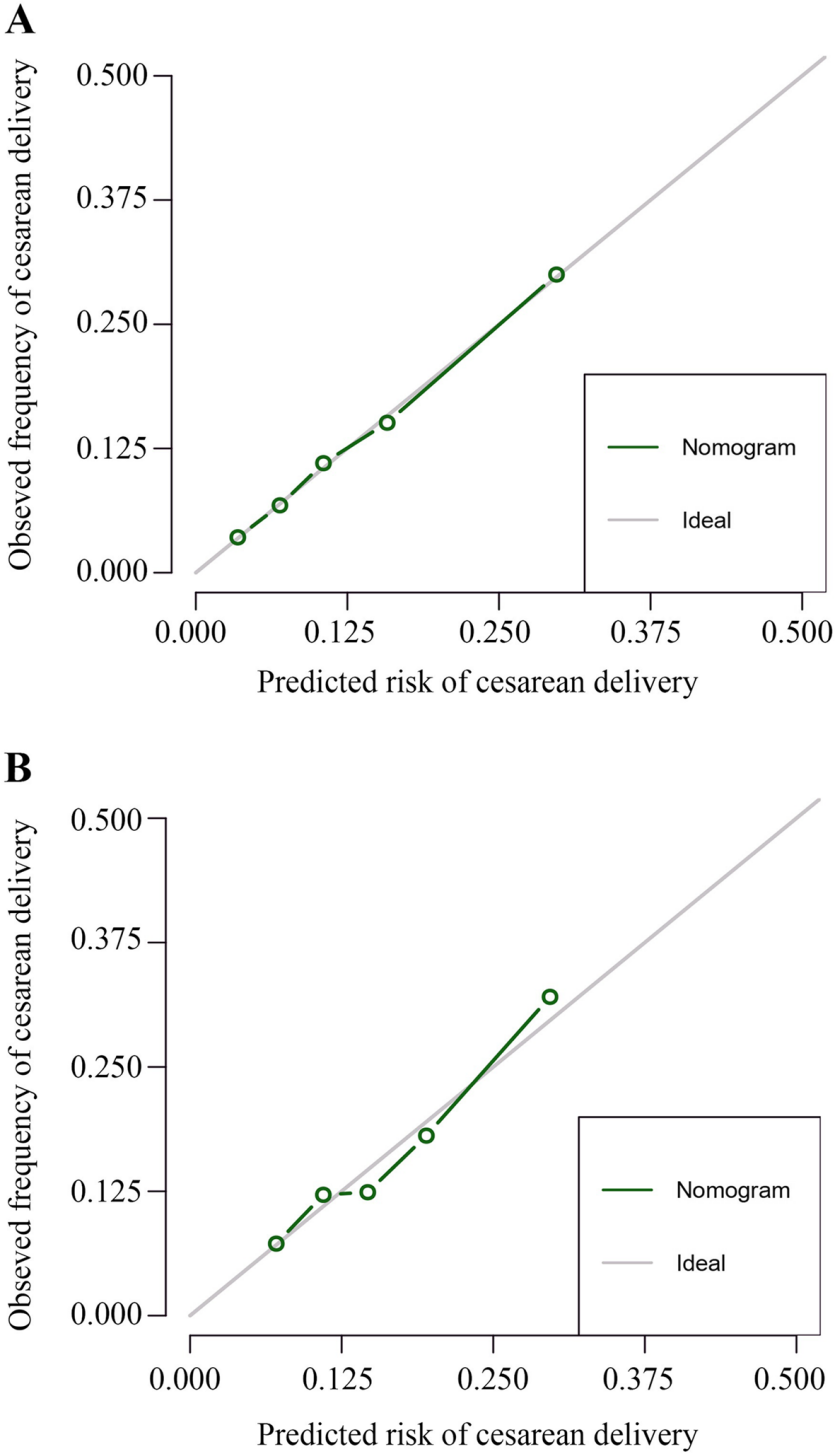
Calibration curve for the final multivariable model depicted in the nomogram. The calibration curve for predicting risk of cesarean delivery among nulliparous at term after induction of labor in **A** derivation cohort and **B** validation cohort. The grey line indicates the ideal reference line where the predicted probabilities estimated from the model would match the observed proportion of cesarean delivery. Nomogram-predicted probability is plotted on the x-axis, and the actual observation proportion is plotted on the y-axis

The nomogram was created based on coefficients of parameters enrolled in the final logistic regression (Fig. 1). The receiver operating characteristic curve for the nomogram achieved an area under the curve (AUC) of 0.73 (95%CI, 0.70-0.75) for internal validation and 0.67 (95%CI, 0.64-0.702) for external validation after 1000 bootstrap resampling. The sensitivity and specificity of the nomogram reached 0.63 and 0.71, respectively. The calibration curve for both derivation and validation cohort revealed good agreement between predicted risk of cesarean delivery after induction of labor by the nomogram and actual observation, as shown in Fig. 2A and B.

The initial literature review revealed 271 hit. After reading titles and abstracts, 18 articles on cesarean delivery after induction of labor were identified. Fifteen literatures were excluded due to various reasons. Details on literature selection were presented in Fig. S1.

**Fig. 3:**
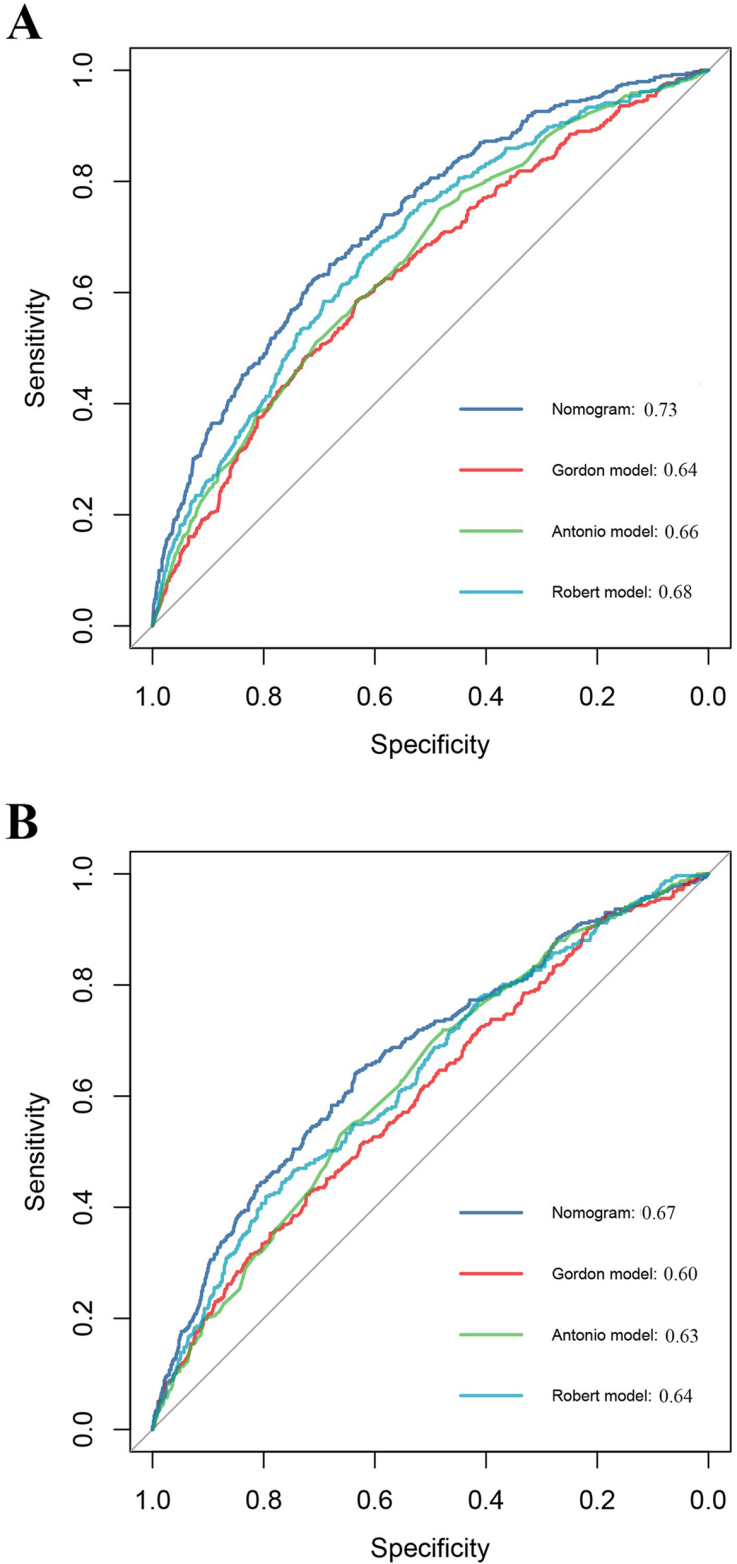
Receiving operating curve (ROC) of the nomogram and existing models. ROC of the nomogram and existing models for derivation cohort (**A**) and validation cohort (**B**). AUC of the nomogram reaches 0.73 in derivation cohort, which is significantly higher than three existing models (*P*<0.05). Meanwhile, AUC the nomogram reaches 0.67 in validation cohort, which is also significantly higher than three existing models (*P*<0.05)

The remaining 3 models were finally included in our study [18–20]. The probability of cesarean delivery after induction of labor for each patient was calculated according to the mathematical equation published in article. The areas under the curve were 0.68, 0.66 and 0.64 for Robert model, Antonio model and Gordon model, respectively, which were significantly lower than AUC of our nomogram in derivation dataset (Fig. 3A). Meanwhile, similar results were found when applying validation dataset to these models (Fig. 3B).

## Discussion

In this single-institutional retrospective cohort of high-risk nulliparous women who underwent induction of labor, we found that maternal age, height, uterine height, abdominal circumference, estimated fetal weight, initial station, initial cervical effacement and initial cervical consistency were independent risk factors for cesarean delivery. Besides, indications for induction of labor also independently affected the rate of cesarean delivery. Using these factors, a nomogram was developed and validated to calculate the likelihood of cesarean delivery, which achieved acceptable AUC of 0.73 for internal validation and 0.67 for external validation. Further analysis revealed that the nomogram showed better discriminative ability than three models published previously.

The risk factors enrolled in the nomogram have been previously reported in literatures. In consistent with previous studies, maternal age and height were associated with cesarean delivery [10, 20–23]. Meanwhile, medical indications for induction of labor, such as PROM, late term, diabetes mellitus and hypertensive disorder of pregnancy were also shown to be independently affected rate cesarean delivery [10, 23]. In our research, uterine height, abdominal circumference and estimated fetal weight were modifiable risk factor, influencing probability of cesarean delivery. Traditionally, Bishop score has been used as the standard evaluation for induction planning. However, not all components were related to cesarean delivery [10, 23]. Cervical dilation, a favorable factor for vaginal delivery, was not recognized as a risk factor in our study. This was likely due to the fact that only a few patients were enrolled with dilated cervix. Meanwhile, cervical position was not associated with cesarean delivery after adjusting for confounders in the large cohort.

Efforts have been made to assess the risk of cesarean delivery among different populations and AUC of these models ranged from 0.68-0.79. Gordon et al. established a prediction model for cesarean delivery after labor induction in nulliparous by four risk factors, including maternal age, height, gestational age and fetal sex [18]. The AUC were 0.68 for internal validation and 0.67 for external validation, which seemed lower than our nomogram. Meanwhile, models published by Antonio Hernández-Martínez and Robert. M Rossi revealed better AUC of 0.77 (95%CI 0.73-0.80) and 0.79 (95%CI 0.764-0.802) for both nulliparous and multiparous women after induction of labor, respectively [19, 20]. The differences might result from population composition, since multiparous women were more likely to experience successful vaginal delivery. Therefore, we chose to emphasize on nulliparous women as efforts to reduce primary cesarean delivery regarding its contribution to cesarean delivery rate.

To further compare the discriminative ability of our nomogram and three models mentioned previously, we performed a validation process, using both derivation dataset and validation dataset. AUC of our nomogram reached 0.73 for internal validation and 0.67 for external validation, which showed better accuracy. Therefore, our nomogram might be more specialized for nulliparous in Chinese population.

Our study aimed to establish a nomogram predicting probability of cesarean delivery after induction of labor. The nomogram achieved clinical useful prediction of cesarean delivery by basic characteristics. However, we should aware that the nomogram is constructed to help with patient consultation instead of making clinical decision directly. For example, a nulliparous woman of 30 years (26 points) with hypertensive disorder (22 points) is in her 37th week of pregnancy. Height of the patient is 155 cm (63 points), with 30 cm (6 points) of uterine height and 100 cm (26 points) of abdominal circumference, and the estimated fetal weight is 3000 g (18 points). The cervical condition is unfavorable [consistency, firm (21 points); station, − 3 (17 points); effacement (%), 0-30 (56)]. The total point reaches 255, with an estimated probability of cesarean delivery of 78% after induction of labor. The chance of cesarean delivery is extremely high and the women could have chosen to expect a spontaneous labor if there is no strong indication for induction.

Because induction of labor will continue to be one important method for pregnancies at risk of maternal and neonatal morbidity, future studies should focus on patients with high risk of cesarean delivery to choose the safest option.

Our results were robust for several reasons. First, we used large, well-described datasets from one retrospective study on changes of cesarean delivery rate by 10-Group Classification System to establish the nomogram. It provided data with high quality. Secondly, variables enrolled in the nomogram could be easily measured antepartumly. Hence, the nomogram will be easy and inexpensive to daily clinical application. Importantly, both internal and external validation of the nomogram was carried out to ensure its reproducibility in more generalized population. Meanwhile, better discriminative ability of the nomogram was revealed by comparison with three existing models.

The main limitation of the study was the retrospective design. Potential predictors, like pre-pregnancy weight, were not taken into account, because information of these characteristics was not documented in our medical records. Second, the application of the nomogram should be limited to patients who met inclusion criteria of the research. For example, patients with elective induction of labor should not be consulted by the nomogram, since the nomogram was constructed from nulliparous with medical indications for induction of labor. Third, external validation of data from other institute should be carried out due to the single institute nature of our study. Besides, relationships between different characteristics and indications of cesarean delivery should be further evaluated to improve the nomogram.

## Conclusion

We constructed a nomogram for cesarean delivery after induction of labor in nulliparous women. The nomogram performed well in both internal and external validation. By providing probability of cesarean delivery after induction of labor, our nomogram would help to consult with delivery mode before undergo of this obstetric procedure.

## Supplementary Information

**Additional file 1: Supplementary Figure 1.** Flowchart of literature review of existing models for labor induction. Flowchart of literature review of existing models for labor induction in a two-step process. A total of 253 articles are excluded after reading titles and abstracts, and 15articels are excluded after going though full text.

## Data Availability

The datasets used and/or analyzed during the current study are available from the corresponding author on reasonable request.

## Abbreviations

PROM: Premature rupture of membrane
ROC: Receiver-operative characteristics
AUC: Area under the curve
CI: Confidence interval
OR: Odds ratio
SD: Standard deviation
wk: Week
