# Supplementary figures and images for: Nomogram predicting cesarean delivery undergoing induction of labor among high-risk nulliparous women at term: a retrospective study
Source: BMC Pregnancy Childbirth. 2022 Jan 21;22:55. doi: 10.1186/s12884-022-04386-8 (PMC8783481; doi:10.1186/s12884-022-04386-8)

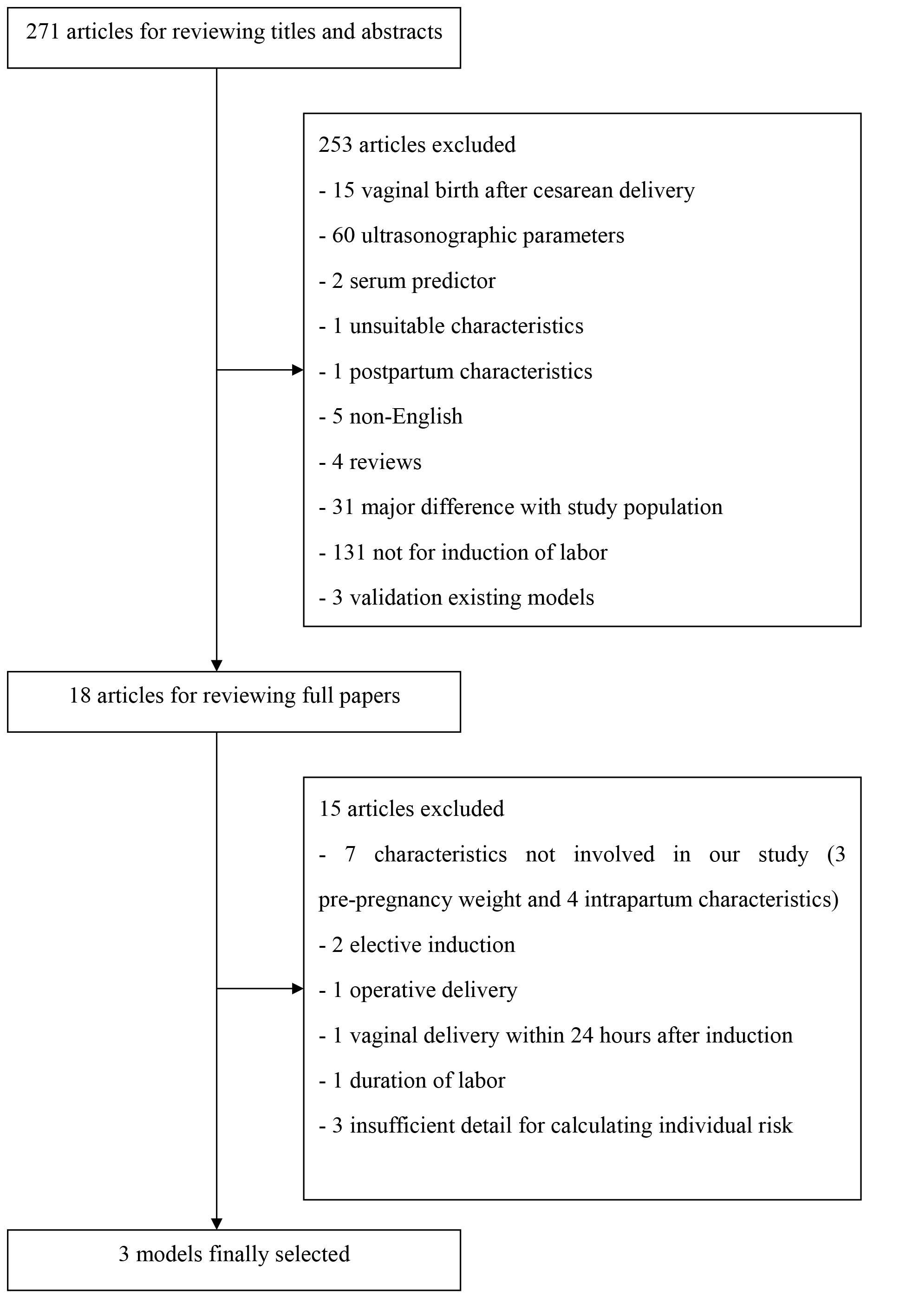

Supplement: Supplementary file 1 — Additional file 1: Supplementary Figure 1. Flowchart of literature review of existing models for labor induction. Flowchart of literature review of existing models for labor induction in a two-step process. A total of 253 articles are excluded after reading titles and abstracts, and 15articels are excluded after going though full text. [file 12884_2022_4386_MOESM1_ESM.png]
